# Exploring the potential of machine learning and magnetic resonance imaging in early stroke diagnosis: a bibliometric analysis (2004–2023)

**DOI:** 10.3389/fneur.2025.1505533

**Published:** 2025-03-14

**Authors:** Jian-cheng Lou, Xiao-fen Yu, Jian-jun Ying, Da-qiao Song, Wen-hua Xiong

**Affiliations:** Yiwu Hospital of Traditional Chinese Medicine, Yiwu, China

**Keywords:** stroke, machine learning, magnetic resonance imaging, bibliometric analysis, WoSCC

## Abstract

**Objective:**

To examine the focal areas of research in the early diagnosis of stroke through machine learning identification of magnetic resonance imaging characteristics from 2004 to 2023.

**Methods:**

Data were gathered from the Science Citation Index-Expanded (SCI-E) within the Web of Science Core Collection (WoSCC). Utilizing CiteSpace 6.2.R6, a thorough analysis was conducted, encompassing publications, authors, cited authors, countries, institutions, cited journals, references, and keywords. This investigation covered the period from 2004 to 2023, with the data retrieval completed on December 1, 2023, in a single day.

**Results:**

In total, 395 articles were incorporated into the analysis. Prior to 2015, the annual publication count was under 10, but a significant surge in publications was observed post-2015. Institutions and authors from the USA and China have established themselves as mature academic entities on a global scale, forging extensive collaborative networks with other institutions. High-impact journals in this field predominantly feature in top-tier publications, indicating a consensus in the medical community on the application of machine learning for early stroke diagnosis. “deep learning,” “magnetic resonance imaging,” and “stroke” emerged as the most attention-gathering keywords among researchers. The development in this field is marked by a coexisting pattern of interdisciplinary integration and refinement within major disciplinary branches.

**Conclusion:**

The application of machine learning in the early prediction and personalized medical plans for stroke patients using neuroimaging characteristics offers significant value. The most notable research hotspots currently are the optimal selection of neural imaging markers and the most suitable machine learning algorithm models.

## Introduction

Stroke is an acute cerebrovascular disorder, precipitates enduring cerebral damage, disability, and even mortality upon its onset (1–3). Studies have identified it as the second leading cause of death worldwide (4). Notably, 11% of stroke survivors experience a recurrence within a year, and 39% within a decade (5). Generally, strokes arise either from blood flow obstruction (ischemic strokes, constituting 87%) or intracerebral hemorrhage (hemorrhagic strokes, accounting for 10%) (6, 7). Regardless of the type, prompt medical intervention is crucial, as early diagnosis and treatment significantly influence the outcome.

Magnetic resonance imaging (MRI) stands as the gold index in stroke diagnosis (8–10), boasting high temporal and spatial resolution capabilities that enable meticulous observation of subtle cerebral vascular changes (11, 12). Moreover, the analytical methods derived from multimodal MRI data facilitate a nuanced identification of cerebral structural and functional network regulations (13, 14). Hence, the objective visualization tools provided by MRI technology are instrumental in the early diagnosis of stroke. However, many patients fail to adhere to medical advice for regular MRI follow-ups, leading to acute stroke episodes (15, 16). Consequently, there’s an urgent need in the medical field for a sophisticated neuroimaging algorithm capable of early stroke prediction, mitigating the issue of clinical data scarcity due to patient non-compliance.

Machine learning (ML) algorithms can automate the interpretation of abnormal imaging patterns, accelerating the diagnostic process in urgent scenarios (17–19). They integrate data from diverse sources, including MRI, clinical records, and vital signs, to assess an individual’s future stroke risk. Additionally, ML enhances the precision and sensitivity of stroke diagnosis, particularly in early stages, by learning from extensive datasets (20). Deep learning, a subset of ML, represents one of the most advanced and specialized approaches within the broader ML framework. While ML encompasses a wide range of algorithms, deep learning focuses on neural network architectures capable of automatically extracting high-level features from complex data. In stroke diagnosis, deep learning has garnered significant attention due to its exceptional performance in processing MRI data, particularly for identifying subtle imaging markers of early stroke. Consequently, the bibliometric analysis in this study focuses on ML as the overarching framework, while acknowledging deep learning as a key contributor to advancements in this field. In recent years, ML has been increasingly applied to early stroke detection, with its reliability validated by authoritative multicentric randomized controlled trials (RCTs) (21) and meta-analysis (22) of high evidentiary value. However, the field of academic collaboration networks, developmental trends, and research frontiers in using ML for early stroke diagnosis through MRI feature recognition still lacks extensive bibliometric research.

CiteSpace software, a visualization tool, qualitatively and quantitatively elucidates the interconnected contributions of authors, regions, institutions, and their collaboration networks (23). Its most notable attribute is the insight into research hotspots and frontier areas, along with predictions on specific field’s future development trajectories. Compared to traditional literature reviews and meta-analyses, the bibliometric analysis facilitated by CiteSpace offers a more profound and insightful perspective (24–26). This study aims to utilize CiteSpace to comprehensively search the WoSCC for relevant literature from the past two decades, conducting a bibliometric analysis on core authors, their collaboration networks, journals, countries, and affiliated academic institutions. This will deepen our understanding of the frontiers and developmental trends in the early diagnosis of stroke using ML to identify MRI characteristics.

## Materials and methods

### Data sources and search strategy

#### Data sources

The data for this study were sourced from the Science Citation Index-Expanded (SCI-E) within the Web of Science Core Collection (WoSCC), a citation-based database that provides detailed citation information and abstracts. This allows for the calculation of bibliometric indicators such as cited authors, journals, impact factors, *h*-indexes, and citation reports. WoSCC encompasses over 12,000 high-quality academic journals spanning more than 250 disciplines, offering a comprehensive collection of interdisciplinary publications.

Compared to databases like PubMed, which focus primarily on biomedical literature and lack citation metrics, WoSCC offers superior bibliometric capabilities, including citation networks, co-authorship relationships, and keyword co-occurrence trends. Current bibliometric tools also do not support multi-database integration due to challenges such as inconsistent data formats and record duplication, complicating the research process and compromising data consistency.

While relying solely on WoSCC may exclude articles indexed in databases like PubMed or Scopus, which could contain relevant studies on machine learning and MRI in stroke diagnosis, WoSCC’s interdisciplinary coverage and citation-based metrics make it suitable for this study. A broad search strategy was applied to minimize dataset bias. Future research could explore multi-database integration as methodologies evolve to address associated challenges.

#### Search strategy

**Figure 1:**
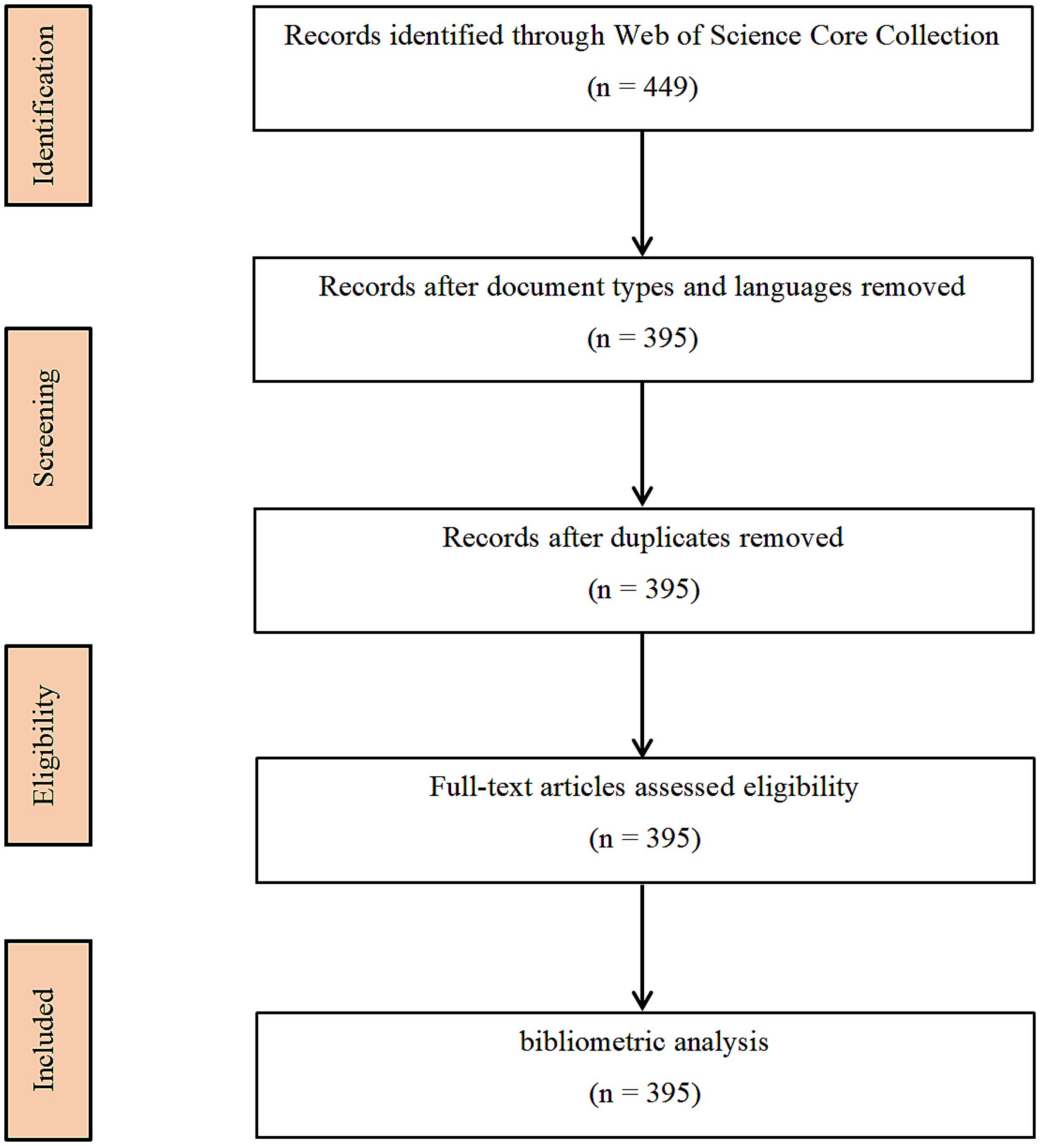
Map of literature screening process related to machine learning and MRI in early stroke diagnosis.

The data retrieval strategy encompassed key topics such as “stroke,” “magnetic resonance imaging,” and “machine learning” (Figure 1). This encompassed a nearly two-decade span of publications, from December 1, 2004, to December 1, 2023, with the retrieval completed within a single day, December 10, 2023. We imposed no geographical restrictions on the publishing countries, but required the language to be English and the research type to be “article” (27–29). Details of the retrieval strategy and results are provided in Table 1. A total of 395 articles were identified, which, after importing into CiteSpace and eliminating duplicates, confirmed the absence of redundancies.

**Table 1:** The topic search query.

Set	Results	Search query
#1	205,292	TS = ((stoke) OR (brain infarction) OR (cerebrovascular) OR (cerebral infarction))
#2	909,543	TS = ((machine learning) OR (deep learning) OR (artificial intelligence) OR (machine intelligence) OR (neural network) OR (natural language processing) OR (hybrid intelligent system) OR (CNN) OR (LSTM) OR (RNN))
#3	588,300	((Magnetic Resonance Imaging) OR (Neuroimaging) OR (MRI))
#4	449	#1 AND #2 AND #3
#5	395	#4 AND Article (Document Types) AND English (Languages)

Web of Science Core Collection (December 1 2004 to December 1, 2023).

### Analysis tool

The visualizations generated by CiteSpace 6.2.R6 typically include nodes, links, colors, clusters, and timelines. Nodes usually represent various research papers, authors, journals, or keywords, with the size of a node often indicating its significance or impact, such as citation frequency. Links denote the relationships between nodes, like citation or collaboration connections, with the thickness of a line possibly indicating the strength or frequency of the relationship. Different colors may represent different time periods or various research fields or categories. Clusters, composed of closely connected nodes, signify specific research themes or areas, aiding in understanding the primary branches and trends within a research field. The timeline exhibits the evolution of keywords or themes over time. Interpreting these visualizations aids in uncovering hot topics, developmental trends, and relationships in research concerning the application of ML in the field of stroke.

The parameters used in CiteSpace 6.2.R6 were as follows: time slices covered the period from 2004 to 2023, with each slice representing 1 year. All terms were included, such as “title,” “abstract,” “author keywords,” and “keywords plus.” To enhance the clarity of the final visualizations and facilitate the observation of relationships between publications, we set the *g*-index’s *k*-value to 50 and employed the Pathfinder algorithm (30, 31).

## Results

### Annual publications

**Figure 2:**
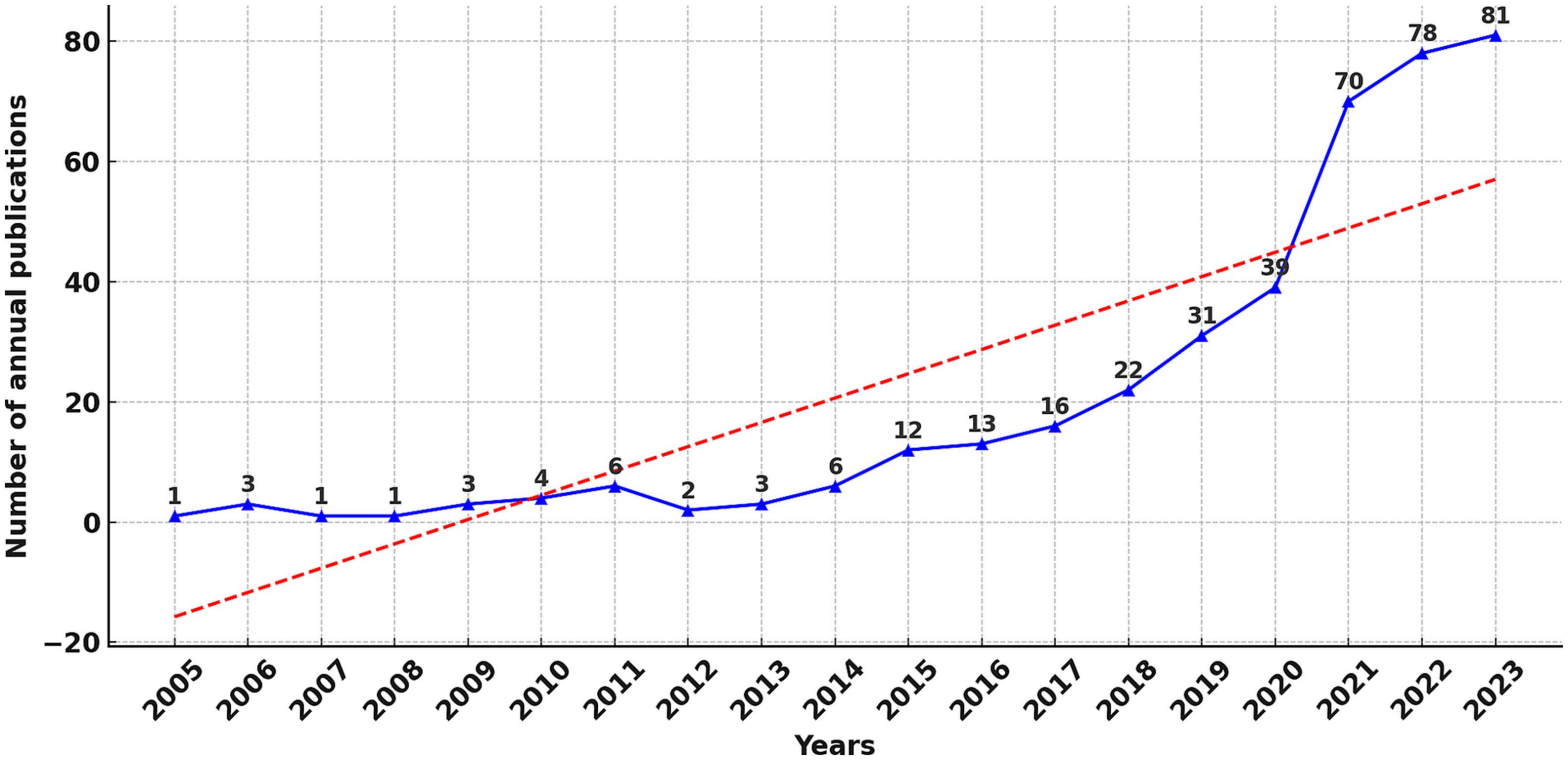
Map of annual publications related to machine learning and MRI in early stroke diagnosis.

Figure 2 illustrates the annual publication trend in using ML to analyze MRI characteristics for the early diagnosis of stroke. It was observed that prior to 2015, the quantity of publications remained at a relatively low level, correlating significantly with the nascent phase of ML as an emerging discipline. From 2015 onwards, there has been a substantial increase in publications, attributed to advancements in the computational capabilities of ML and the refinement of algorithmic architectures. These developments have shown promise in enhancing the accuracy of diagnosing stroke, its subtyping, and prognostic predictions (32). Our investigation revealed that in 2015, the U.S. Food and Drug Administration (FDA) approved several ML-based medical devices, such as RapidAI^®^ and Viz.ai^®^, which have played a pivotal role in the early diagnosis and treatment decision-making of strokes. The trajectory of the trend line leads us to infer that in the next 5–10 years, a new peak in the volume of publications is likely to emerge.

In addition to describing publication trends, the correlation analysis highlights a moderate positive relationship between the emergence of machine learning and its application in stroke research. This result indicates that advancements in ML directly influenced its adoption in stroke diagnosis and treatment. For example, the spike in publications after 2015 aligns with the FDA approval of ML-based medical tools, such as RapidAI^®^ and Viz.ai^®^, which are designed to enhance diagnostic workflows.

### Analysis of authors

**Figure 3:**
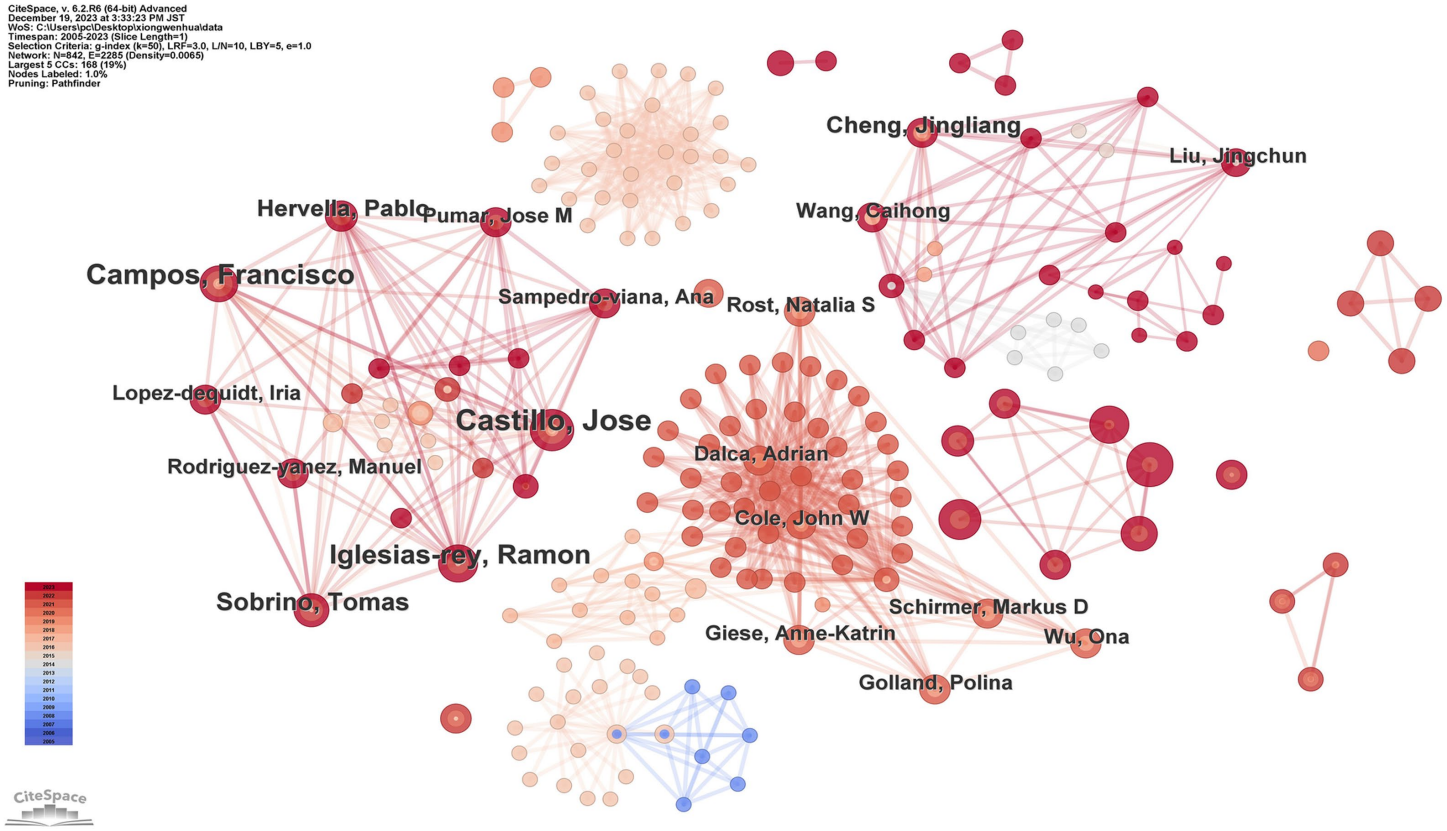
Map of authors related to machine learning and MRI in early stroke diagnosis.

Figures 3 and Table 2 display the authorship information behind the 395 published articles. Each node represents an author, with the connecting lines indicating collaborative relationships between them. The top 10 authors, in descending order, are: Castillo, Jose (9 publications); Wang, Yongjun (9 publications); Jing, Jing (8 publications); Campos, Francisco (8 publications); Meng, Xia (7 publications); Iglesias-rey, Ramon (7 publications); Sobrino, Tomas (6 publications); Li, Zixiao (6 publications); Chen, Cheng (5 publications); Zhao, Xingquan (5 publications). It was observed that all of the top 10 authors hail from Spain and China. This pattern reflects the strong research infrastructure and significant investments in ML and medical research within these countries. For instance, Spain has long been recognized for its clinical stroke research expertise, while China has emerged as a leader in applying ML technologies to medical diagnostics due to its large patient datasets and growing interdisciplinary collaborations.

**Table 2:** Top 10 authors related to machine learning and magnetic resonance imaging in early stroke diagnosis.

Rank	Author	Frequency	Year	Country
1	Castillo, Jose	9	2016	Spain
2	Wang, Yongjun	9	2022	People’s Republic of China
3	Jing, Jing	8	2022	People’s Republic of China
4	Campos, Francisco	8	2016	Spain
5	Meng, Xia	7	2022	People’s Republic of China
6	Iglesias-rey, Ramon	7	2016	Spain
7	Sobrino, Tomas	6	2016	Spain
8	Li, Zixiao	6	2022	People’s Republic of China
9	Chen, Cheng	5	2020	People’s Republic of China
10	Zhao, Xingquan	5	2022	People’s Republic of China

The collaboration network reveals that higher node degree correlates strongly with author centrality, suggesting that prominent authors often serve as key hubs in multi-center studies. For instance, Jose Castillo and Yongjun Wang exhibit significant influence in coordinating international collaborations, reflecting their pivotal roles in advancing research in this domain.

### Analysis of countries

**Figure 4:**
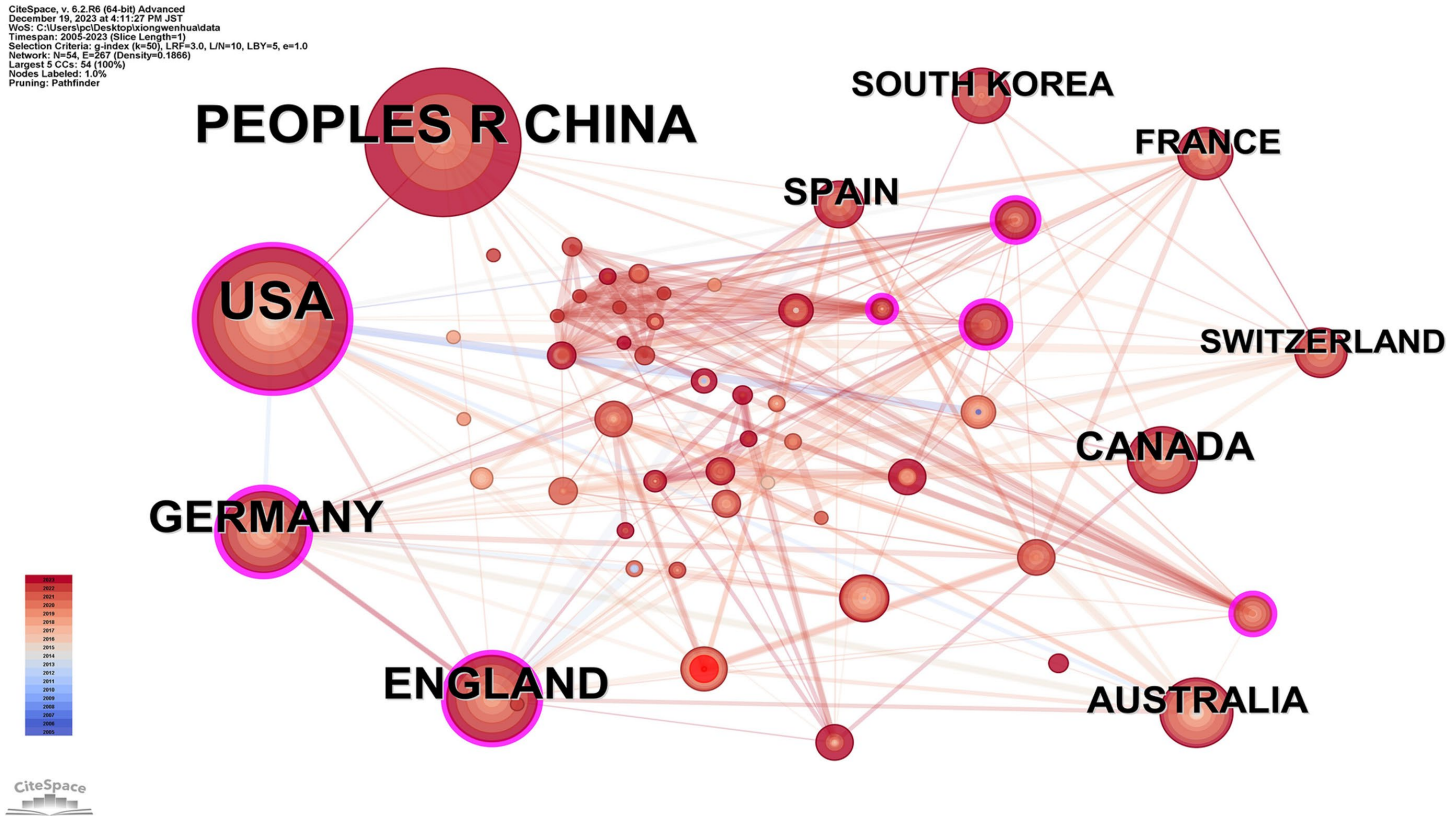
Map of countries related to machine learning and MRI in early stroke diagnosis.

Figures 4 and Table 3 present the collaborative network among countries in this field of research, revealing a network comprising 54 nodes and 267 edges. The top contributors by publication volume are the People’s Republic of China (136); USA (135); England (54); Germany (51); and Canada (34). However, a closer analysis reveals an interesting distinction between publication volume and centrality, which measures the influence of a country within the collaboration network. For example, while China leads in publication volume, its centrality is relatively low, indicating fewer collaborative connections with other nations compared to Germany (centrality: 0.24) and the USA (centrality: 0.14). This discrepancy suggests that while China and the USA dominate in output, Germany plays a more integrative role in fostering international collaborations. Such insights underline the importance of not only the quantity but also the quality and connectivity of research contributions in advancing the field.

**Table 3:** Top 10 frequency and centrality of countries related to machine learning and magnetic resonance imaging in early stroke diagnosis.

Rank	Frequency	Countries	Rank	Centrality	Countries
1	136	People’s Republic of China	1	0.24	Germany
2	135	USA	2	0.19	India
3	54	England	3	0.15	England
4	51	Germany	4	0.15	Netherlands
5	34	Canada	5	0.15	Austria
6	27	Spain	6	0.14	USA
7	25	Australia	7	0.12	Czech Republic
8	20	South Korea	8	0.10	Switzerland
9	19	France	9	0.10	Saudi Arabia
10	18	Switzerland	10	0.07	Canada

In the country network, there is a strong positive correlation between publication volume and collaboration frequency, highlighting the synergy between research activity and international partnerships. Interestingly, the correlation is weaker for centrality, indicating that publication volume does not always reflect the strategic importance of a country within the network. For instance, Germany, with a centrality of 0.24, leads in bridging interdisciplinary collaborations despite ranking fourth in publication volume.

### Analysis of institutions

**Figure 5:**
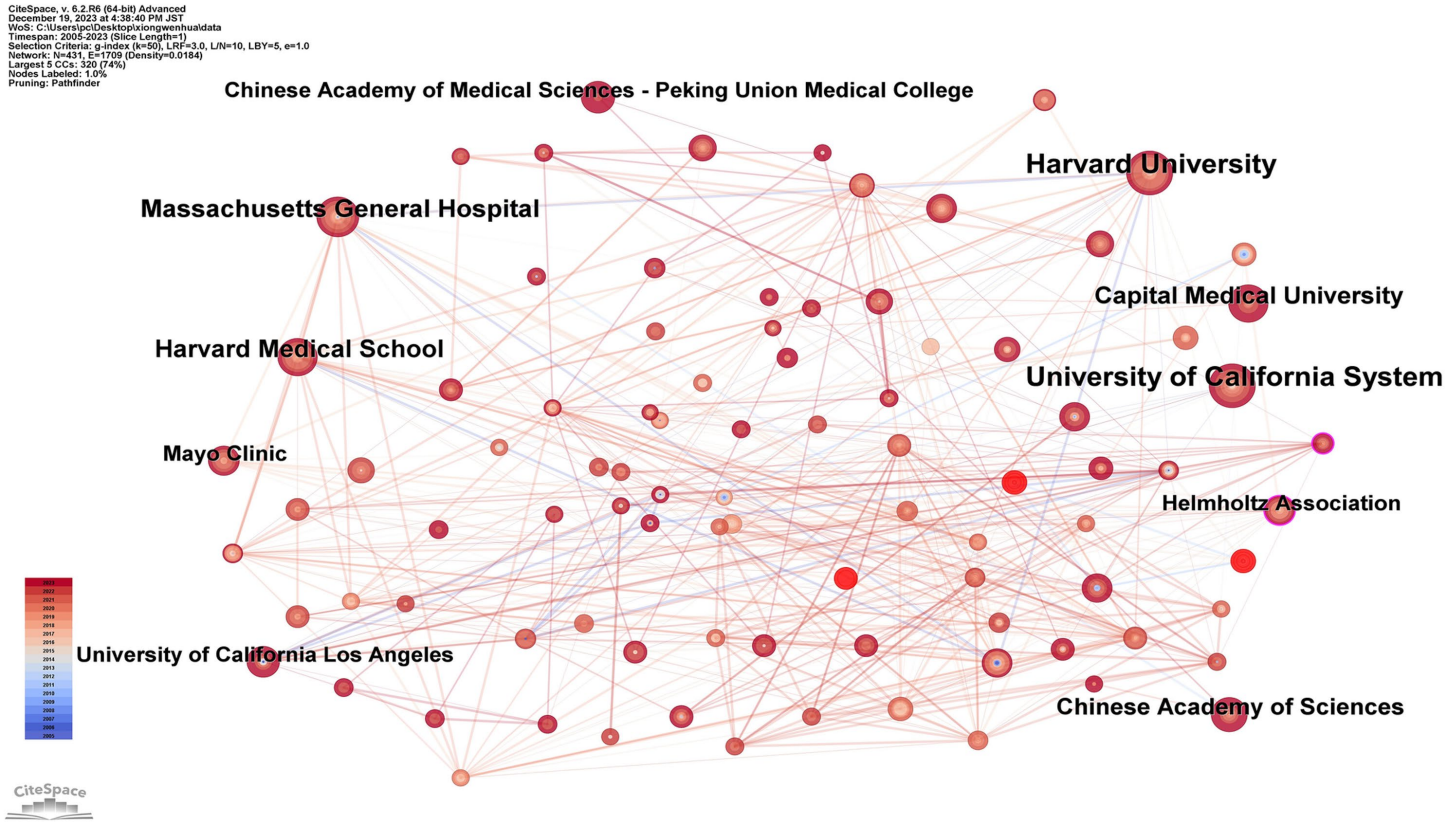
Map of institutions related to machine learning and MRI in early stroke diagnosis.

Figure 5 and Table 4 display the collaborative network of institutions, comprising 431 nodes and 1,709 edges. The top 10 institutions in terms of publication volume are as follows: University of California System (33 publications); Harvard University (33 publications); Massachusetts General Hospital (23 publications); Harvard Medical School (21 publications); Capital Medical University (20 publications); Chinese Academy of Sciences (18 publications); Chinese Academy of Medical Sciences—Peking Union Medical College (14 publications); Helmholtz Association (14 publications); Mayo Clinic (14 publications); University of California Los Angeles (13 publications). It was observed that academic institutions affiliated with the USA dominate the top 10 rankings. The interconnections between institutions across various countries highlight a significant network of collaborations, which is poised to further advance the discipline in this field.

**Table 4:** Top 10 publications of institutions related to machine learning and magnetic resonance imaging in early stroke diagnosis.

Rank	Frequency	Year	Institutions
1	33	2006	University of California System
2	33	2005	Harvard University
3	23	2005	Massachusetts General Hospital
4	21	2006	Harvard Medical School
5	20	2019	Capital Medical University
6	18	2011	Chinese Academy of Sciences
7	14	2022	Chinese Academy of Medical Sciences—Peking Union Medical College
8	14	2012	Helmholtz Association
9	14	2017	Mayo Clinic
10	13	2006	University of California Los Angeles

Institutional collaboration analysis shows a moderate positive correlation between node degree and publication output. Institutions such as the University of California System and Harvard University, which exhibit high node degrees, also demonstrate strong interconnectivity, fostering impactful collaborations that push the boundaries of ML applications in stroke research.

### Analysis of cited journals

**Figure 6:**
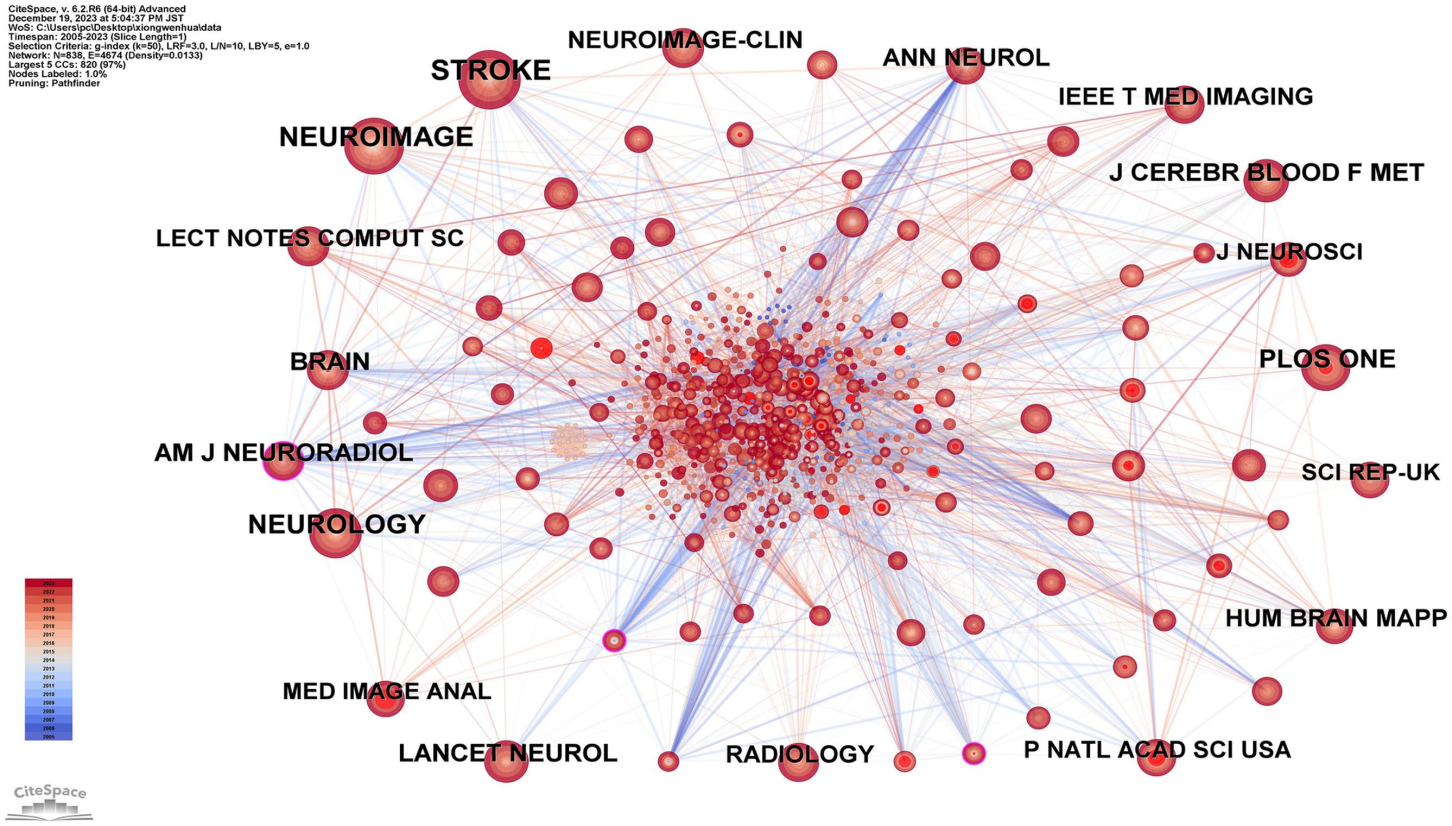
Map of cited journals related to machine learning and MRI in early stroke diagnosis.

Figure 6 and Table 5 showcase the cited journal network, consisting of 838 nodes and 4,674 edges. The top 10 journals by citation frequency are: *Stroke* (248 citations); *NeuroImage* (215 citations); *Neurology* (183 citations); *PLoS One* (145 citations); *Brain* (127 citations); *Lancet Neurol* (125 citations); *J Cereb Blood Flow Metab* (125 citations); *Am J Neuroradiol* (119 citations); *Ann Neurol* (117 citations); *Hum Brain Mapp* (109 citations). Additionally, journals with notable centrality (indicated by purple rings) include *Ann NY Acad Sci* (0.12); *Am J Neuroradiol* (0.11); *Acta Neuropathol* (0.11). These journals primarily cover neurology, neuroimaging, and computer science. For instance, *Stroke* has an impact factor of 8.3, with *NeuroImage* and *Neurology* also being top-tier journals in this field.

**Table 5:** Top 10 frequency and centrality of cited journals related to machine learning and magnetic resonance imaging in early stroke diagnosis.

Rank	Frequency	Cited journals	Rank	Centrality	Cited journals
1	248	Stroke	1	0.12	Ann NY Acad Sci
2	215	NeuroImage	2	0.11	Am J Neuroradiol
3	183	Neurology	3	0.11	Acta Neuropathol
4	145	PLoS One	4	0.09	Ann Neurol
5	127	Brain	5	0.09	Acad Radiol
6	125	Lancet Neurol	6	0.09	IEEE Int Conf Neural Netw Proc
7	125	J Cereb Blood Flow Metab	7	0.08	Am J Cardiol
8	119	Am J Neuroradiol	8	0.07	IEEE Trans Med Imaging
9	117	Ann Neurol	9	0.07	Annu Rev Neurosci
10	109	Hum Brain Mapp	10	0.06	Arch Neurol

The journals Stroke and NeuroImage exhibit the highest normalized citation impact, indicating their influence in bridging neurology and imaging studies. Additionally, metrics such as *h*-index and Eigenfactor score were analyzed for the top-cited journals to further evaluate their academic impact. For instance, Stroke has an impact factor of 8.3 and an *h*-index of 150, showcasing its long-standing relevance in stroke research. Similarly, NeuroImage demonstrates a significant *h*-index of 230, reflecting its importance in neuroimaging and machine learning studies. The cited journal analysis, complemented by impact metrics, highlights the interplay between foundational stroke research and emerging machine learning methodologies. This integrative approach provides robust evidence of the academic networks and key journals shaping this interdisciplinary field.

### Keywords co-occurrence and citation burst analysis

**Figure 7:**
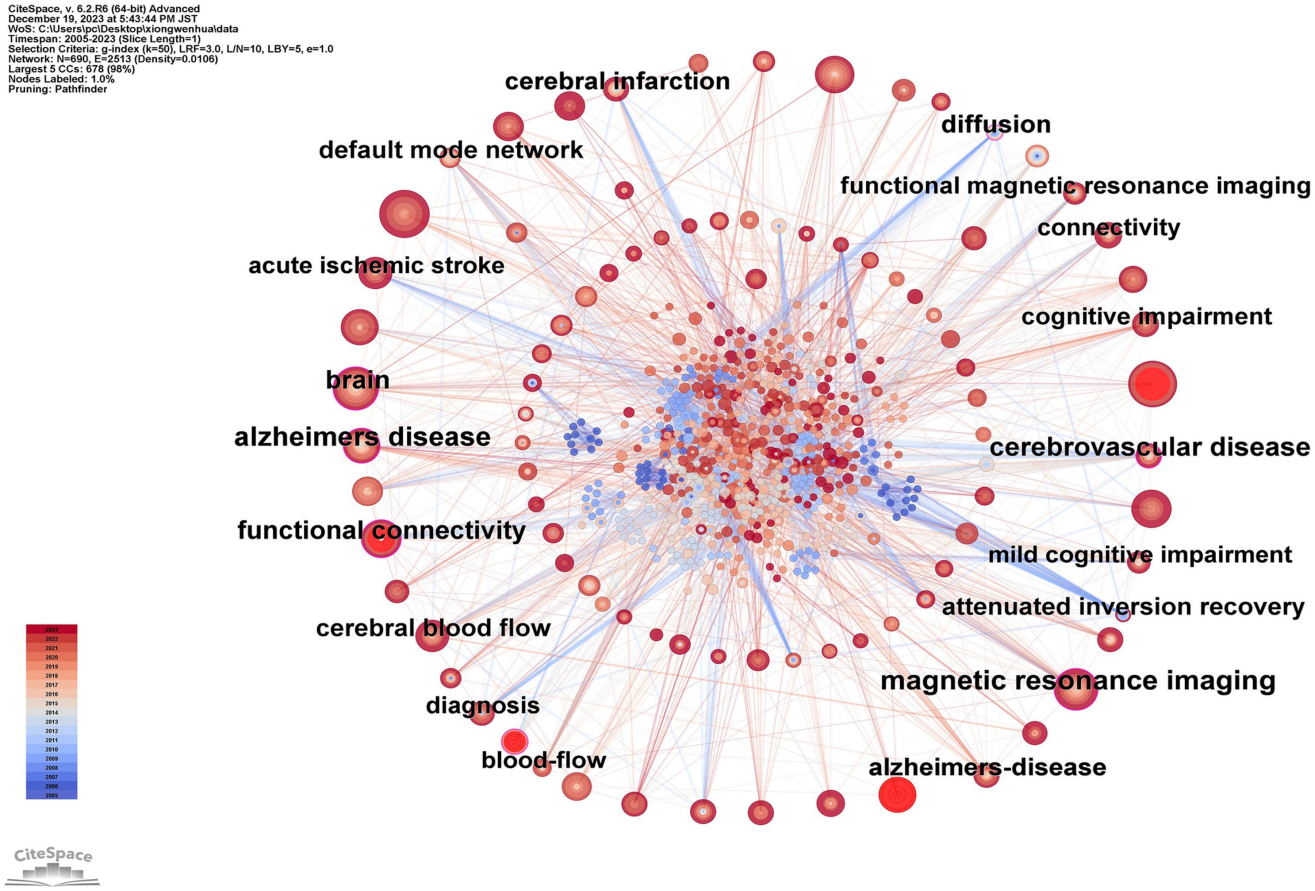
Map of keywords related to machine learning and MRI in early stroke diagnosis.

Figure 7 and Table 6 depict the network graph of keywords, encompassing 690 nodes and 2,513 edges. The top 10 keywords are: deep learning (46 occurrences); machine learning (45 occurrences); brain (39 occurrences); magnetic resonance imaging (36 occurrences); stroke (31 occurrences); MRI (25 occurrences); functional connectivity (25 occurrences); risk (24 occurrences); ischemic stroke (24 occurrences); Alzheimer’s disease (23 occurrences). Analyzing the frequency and centrality of these keywords reveals that “deep learning,” “magnetic resonance imaging,” and “stroke” have emerged as prominent themes in this research area.

**Table 6:** Top 10 frequency and centrality of keywords related to machine learning and magnetic resonance imaging in early stroke diagnosis.

Rank	Frequency	Keywords	Rank	Centrality	Keywords
1	46	deep learning	1	0.19	Alzheimer’s disease
2	45	machine learning	2	0.18	magnetic resonance imaging
3	39	brain	3	0.16	cerebrovascular disease
4	36	magnetic resonance imaging	4	0.15	brain
5	31	stroke	5	0.13	functional connectivity
6	25	MRI	6	0.11	functional MRI
7	25	functional connectivity	7	0.11	diffusion
8	24	risk	8	0.09	cerebral blood flow
9	24	ischemic stroke	9	0.09	Alzheimer’s disease
10	23	Alzheimer’s disease	10	0.09	functional magnetic resonance imaging

**Figure 8:**
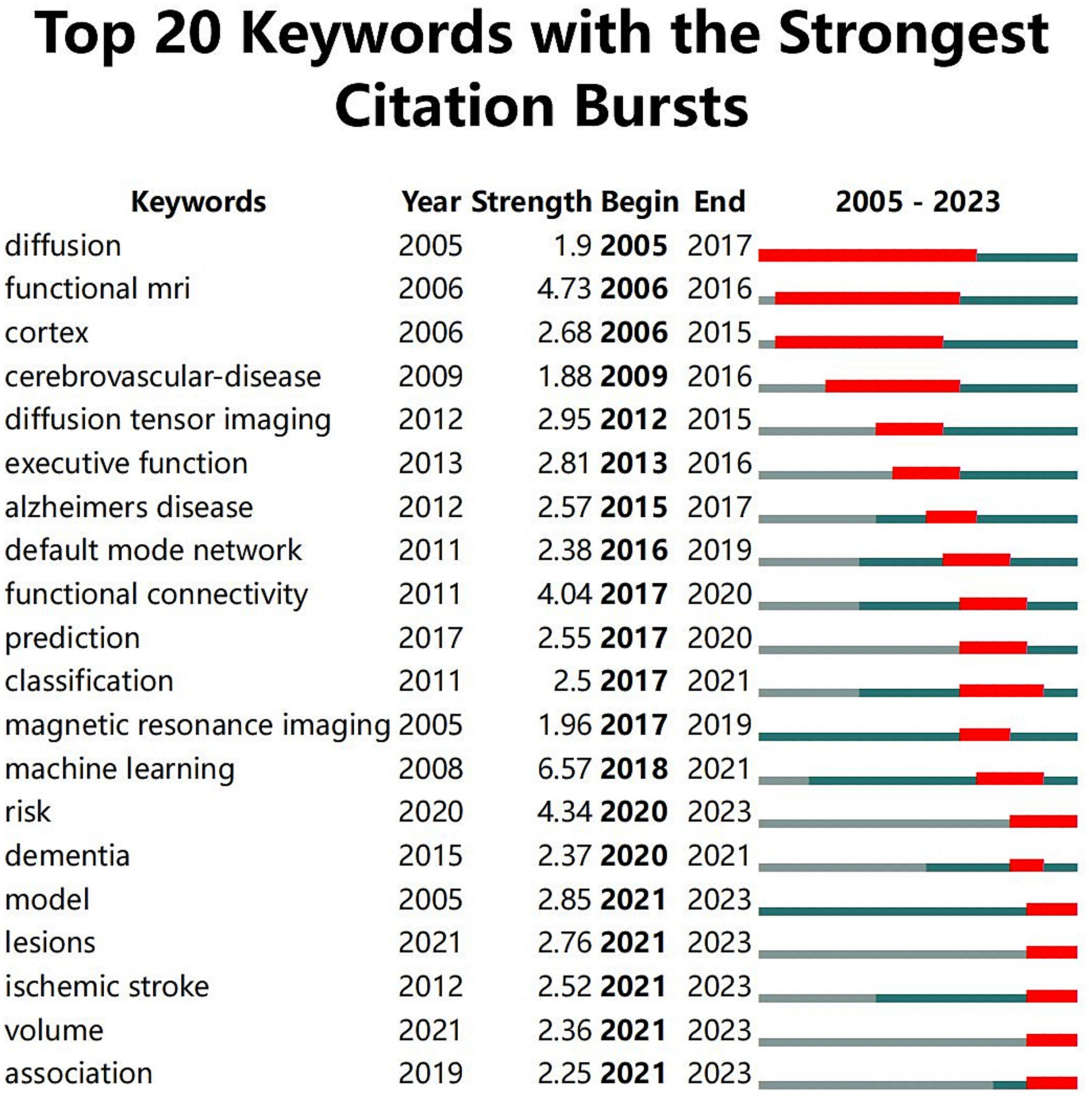
Top 20 keywords with the strongest citation bursts.

Figure 8 illustrates the top 20 keywords with the most robust citation bursts. The beginning and end of each burst are, respectively, marked as “Start” and “End,” with the increase in influence correlating with the rise in the “Strength” value. The pale blue region delineates the study period, while the red portion signifies the start and peak of the bursts. It was observed that “machine learning” exhibited the highest burst strength, reaching 6.57. Furthermore, early attention to “functional MRI” and “diffusion tensor imaging” indicates that changes in brain structure and function had been applied in this field from an early stage. Mid-period keywords like “executive function” and “default mode network” experienced high burst rates, signifying researchers’ growing focus on the interconnections between deep brain networks. In later periods, the frequent emergence of terms such as “prediction,” “classification,” and “machine learning” underscores the extensive application of ML in recent years for the early diagnosis of stroke and the development of individualized treatment plans to prevent the high mortality associated with acute stroke incidents.

The co-occurrence network reveals a strong positive correlation between keyword centrality and burst strength. Keywords such as “deep learning” and “machine learning” not only occur frequently but also drive significant citation bursts, underscoring their pivotal roles in shaping the field. Additionally, the temporal analysis suggests that early bursts in keywords like “functional MRI” paved the way for mid-period focuses on “executive function” and later trends emphasizing “classification” and “prediction.”

### Keywords timeline

**Figure 9:**
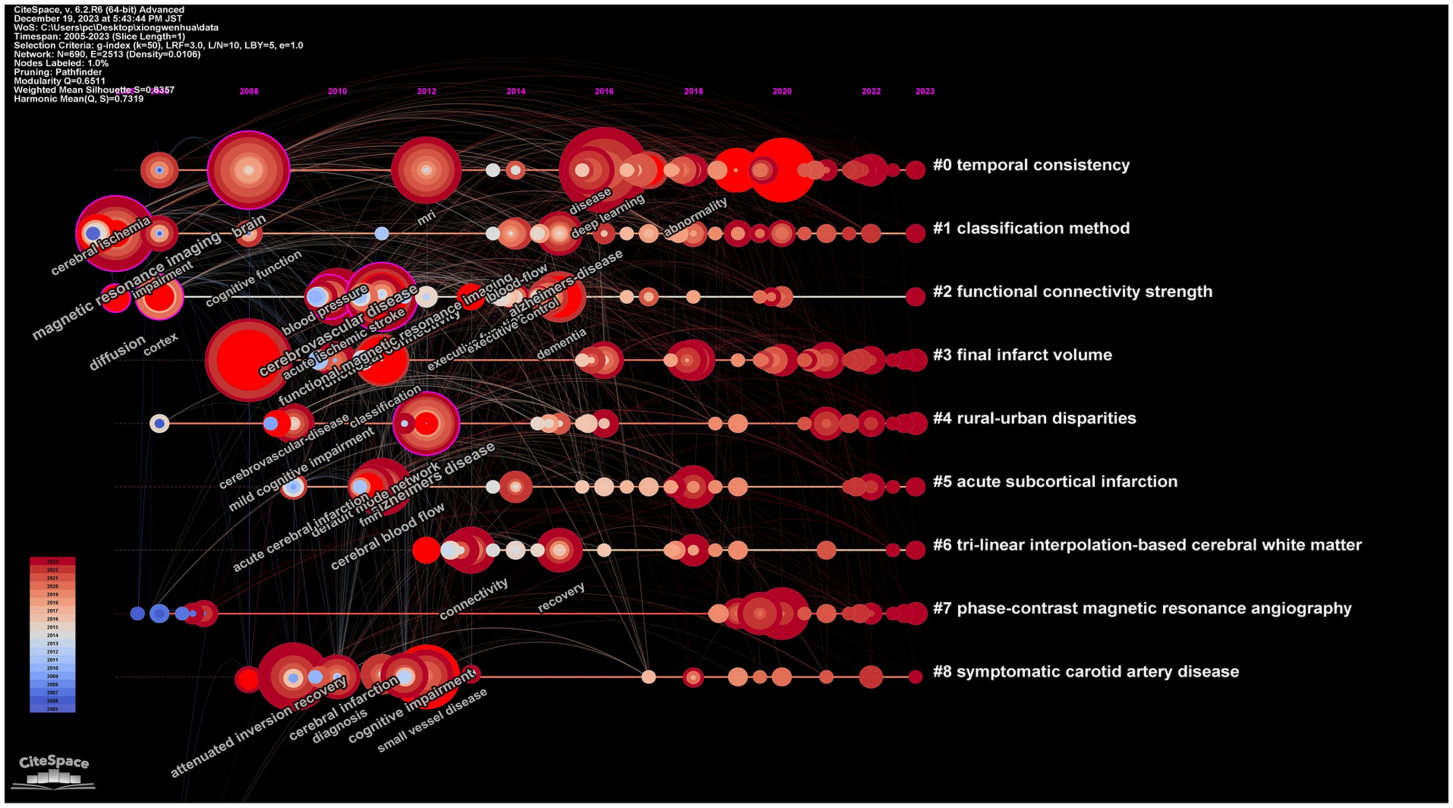
Map of keywords timeline related to machine learning and MRI in early stroke diagnosis.

Figure 9 presents the evolution and interconnections of keywords, arranged chronologically. The timeline extends from left to right, delineating the emergence and disappearance of research keywords from 2004 to 2023. Additionally, the diagram clusters various themes. A total of nine clusters (#0 to #8) are depicted. The first cluster (#0), labeled “temporal consistency,” focuses on topics like deep learning, automated WMH detection, and amorphous object segmentation. The second cluster (#1), identified as “classification method,” concentrates on magnetic resonance imaging, cerebral blood flow, and related themes. The third cluster is marked as “functional connectivity strength,” highlighting areas such as intrinsic functional connectivity and graph theory analysis. The fourth cluster, labeled “final infarct volume,” focuses on chronic venous disease, peripheral artery disease, and similar subjects. The fifth cluster, named “rural-urban disparities,” is centered around risk factors and minority health.

### Cluster dependencies of reference

**Figure 10:**
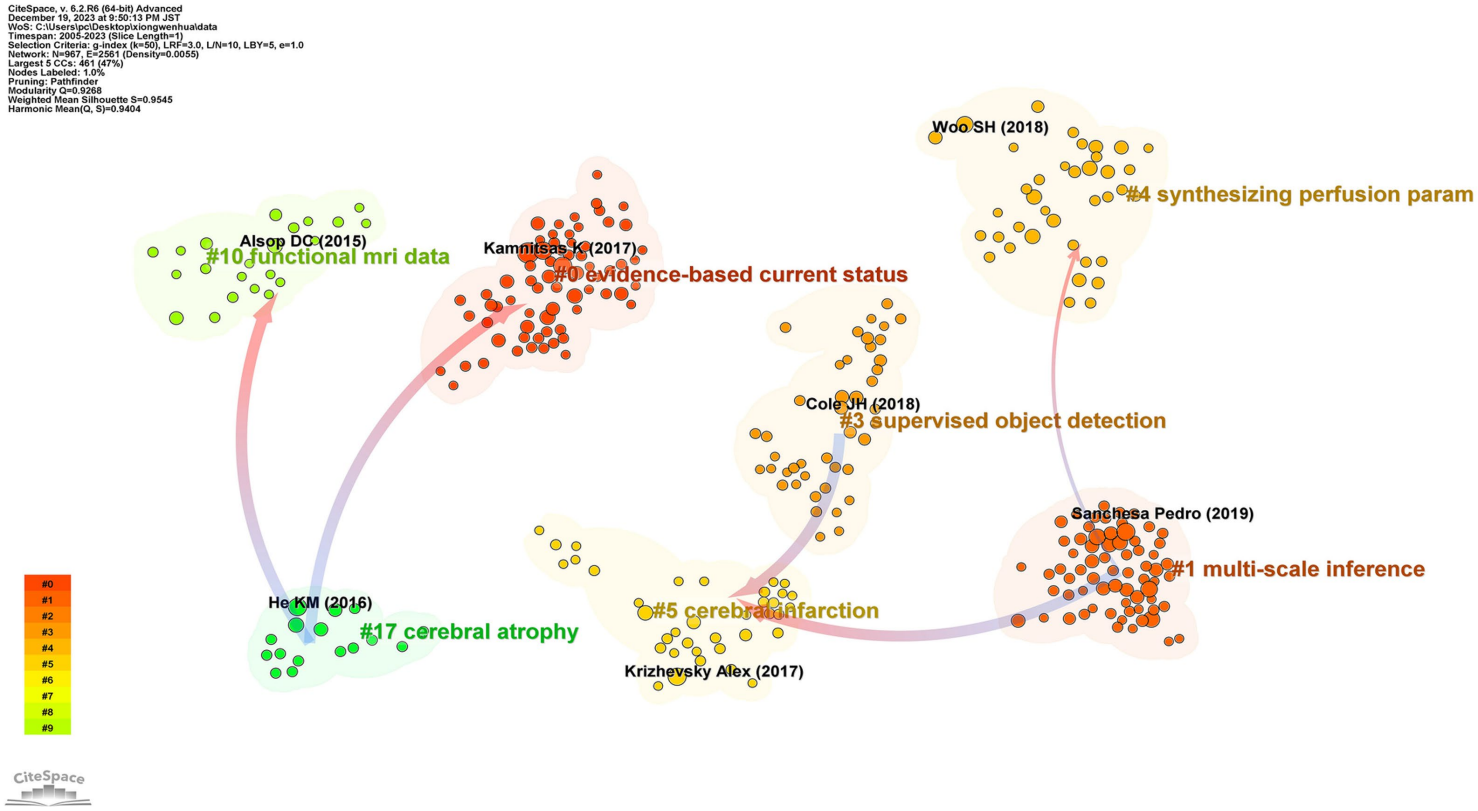
Map of reference of cluster dependencies related to machine learning and MRI in early stroke diagnosis.

Figure 10 showcases the dependency relationships among clusters based on referenced literature. Areas coded in different colors represent distinct clusters of references, while arrows indicate the developmental relationships between these clusters. The convergence of arrows signifies the emergence of new disciplinary branches, while the merging of arrowheads indicates the integration of different disciplines. This is because the tail of an arrow represents the cutting edge of current knowledge, while the head points to the sources of foundational literature.

The developmental pattern in this field primarily exists in a dual form: the coexistence of interdisciplinary integration and the refinement of major disciplinary branches. A detailed analysis reveals several cutting-edge directions of interdisciplinary integration: Neuroimaging and machine learning: The integration of advanced imaging modalities such as diffusion tensor imaging and convolutional neural networks has enabled the development of automated lesion segmentation and early diagnostic models for ischemic stroke. This integration bridges computational algorithms and clinical radiology, advancing both fields. Neurology and bioinformatics: The use of bioinformatics tools in analyzing imaging markers has enhanced the understanding of the molecular underpinnings of stroke. For example, integrating genetic data with MRI-based phenotypes offers new insights into personalized therapeutic strategies. Clinical decision support systems and artificial intelligence: AI-driven CDSS, leveraging ML-based prognostic models, has facilitated real-time decision-making in stroke management, particularly in identifying optimal therapeutic windows. This interdisciplinary collaboration integrates medical informatics, neurology, and computer science. For instance, cluster #17 represents the fusion of neuroimaging and ML (clusters #0 and #10), while cluster #5 reflects the branching of ML applications into specific subdomains of neurology (clusters #1 and #3). These trends underscore the pivotal role of interdisciplinary integration in advancing the field of early stroke diagnosis.

Correlation analysis of the reference dependency network indicates a significant relationship between cluster size and the number of interdisciplinary connections. Larger clusters, such as #0 and #10, exhibit higher connectivity, reflecting their central roles in integrating neuroimaging and ML methodologies. This interdependency highlights the importance of large clusters in driving knowledge transfer and innovation across disciplinary boundaries.

## Discussion

A bibliometric analysis was conducted using CiteSpace, focusing on the early diagnosis of stroke through ML identification of MRI characteristics from 2004 to 2023. This analysis encompassed the collaborative networks of core authors, affiliated institutions, countries, and journals. Comprehensive data were provided, highlighting the focal points and trends in the early diagnosis of stroke using ML to identify MRI characteristics.

### General information

This study illustrates that over the past two decades, a total of 395 publications have been released in the field of early stroke diagnosis using ML to identify characteristics in MRI. The findings show that prior to 2015, the annual number of publications was consistently below 10, reflecting the nascent stage of ML as an academic discipline. Since 2015, there has been a marked increase in publications, a development attributed to the enhanced computational capabilities of ML and the refinement of its algorithmic structures, demonstrating potential in improving the accuracy of stroke diagnosis, subtype classification, and prognostic prediction. The trend line suggests an anticipation of a new peak in publication volume within the next 5–10 years.

An analysis of authors, countries, and their affiliated institutions with higher publication numbers reveals that institutions and authors from the USA and China have established mature academic communities on a global scale, forming extensive collaborative networks with other institutions. This indicates the reliance of ML on the technological level and talent reserves of a country. Notably, despite the lower volume of publications from less developed countries, these nations may still experience high stroke incidence rates.

Through the analysis of interdependencies among clusters in references, it was found that the development pattern in the field of early stroke diagnosis using ML for MRI primarily exists in a form of coexistence between interdisciplinary integration and the refinement of major disciplinary branches. This unique characteristic is likely to promote resource integration, cross-disciplinary idea exchange, and academic innovation within the field.

In summary, ML as an emerging discipline, has shown immense value in early diagnosis of stroke through neuroimaging, medical efficiency, and personalized treatment, with a significant rise in publication volume in recent years. Based on the trends in annual publication volume and innovation in ML algorithms, significant advancements are expected in the next 5–10 years, ultimately aiming to provide precise medical services for stroke patients.

### Research hotspots

Research hotspots in a field are encapsulated by keywords that represent the core content and central themes of studies within that domain. Techniques such as keyword co-occurrence analysis, keyword clustering, and keyword citation bursts enable the monitoring of various emerging trends in a given field. In the realm of using ML for early stroke diagnosis through the identification of MRI features, two primary research hotspots have emerged: the optimal selection of neural imaging markers and the most appropriate ML algorithm models.

### Optimal selection of neural imaging markers

In the pursuit of the most effective neural imaging markers for stroke patients using MRI, researchers primarily focus on cerebral blood flow, brain structure, or brain function. From the perspective of cerebral blood flow, arterial spin labeling (ASL) is a predominant research method. For instance, Liu’s et al. (33) study indicates that the combination of ML and ASL can predict the outcomes of acute ischemic strokes by examining collateral circulation. Regarding brain structure, diffusion weighted imaging (DWI) often serves as the main analytical approach. Yu et al. (34) and Zhu et al. (35), for example, discovered that deep learning using DWI and clinical data is highly sensitive in predicting patients with low-perfusion strokes. From the aspect of brain function, the most valued approach is brain network analysis using functional connectivity (FC) from functional MRI. Li’s et al. (36) findings suggest that multispectral FC variations in brain regions are potential targets for differentiating stroke patients’ recovery and treatment processes. Lu et al. (37) demonstrated that acupuncture could modulate bilateral cerebral hemispheres through distinct targets, restoring abnormal FC and thus facilitating post-stroke motor recovery. Moreover, many studies advocate the integration of multimodal MRI datasets as neural imaging markers, surpassing the predictive accuracy of early stroke onset compared to single-modality data (38–40).

### Optimal machine learning algorithm models

ML algorithms are diverse and continually evolving, with researchers exploring various models for neuroimaging data from MRIs. Pérez Malla et al. (41) and Nishi et al. (42), for instance, regard convolutional neural networks (CNNs) as the most advanced method for early stroke prediction. Billot et al. (43) found that a combination of support vector machines (SVMs) and random forests (RFs) also exhibits commendable performance. Pinto et al. (44) proposed a fully automated deep learning approach encompassing both unsupervised and supervised learning, achieving satisfactory accuracy. Emerging deep learning techniques, such as deep neural networks (DNNs) and reinforcement learning (RL), are gaining traction due to their ability to automatically extract features from raw data and further improve model accuracy. These methods show substantial promise in enhancing early stroke detection by providing deeper, more nuanced insights into complex MRI data patterns. The introduction of these advanced techniques could lead to significant improvements in diagnostic accuracy, particularly in early-stage stroke diagnosis, where subtle changes in brain tissue are often challenging to detect.

Recent advancements in explainable AI (XAI) have also contributed to the interpretability of machine learning models, which is crucial in clinical settings. XAI approaches aim to provide transparent reasoning behind model predictions, enabling healthcare professionals to better understand and trust the automated results. Furthermore, multimodal approaches combining MRI with other data sources, such as genetic or clinical data, are gaining momentum. These methods harness the complementary strengths of different types of data to create more robust models that enhance diagnostic accuracy and prognostic prediction. By integrating various data types, multimodal systems can capture a more comprehensive view of the patient’s condition, improving decision-making in early stroke diagnosis.

The integration of ML with neuroimaging offers significant potential for bridging current gaps in early stroke diagnosis. While traditional methods rely on visual interpretation of MRI scans, ML techniques allow for the automated detection and quantification of subtle patterns that may be overlooked by human evaluators. The ability of ML algorithms to process large volumes of complex MRI data and generate predictive models can improve diagnostic accuracy, particularly in the early stages of stroke, when clinical symptoms may not yet be fully manifest. Furthermore, ML can help to identify imaging markers that correlate with stroke outcomes, offering personalized treatment options for patients. This integration is particularly promising in addressing the challenge of time-sensitive diagnoses, where rapid and accurate assessments can directly impact patient prognosis and recovery. As ML algorithms, including deep learning and multimodal approaches, continue to evolve, their capacity to enhance diagnostic workflows, reduce human error, and accelerate decision-making processes in clinical settings will be invaluable in overcoming the challenges of early stroke diagnosis.

## Conclusion

The application of ML in the early diagnosis, prediction, and individualized medical plans for stroke patients using neuroimaging features offers immense value. This study specifically focused on the role of ML in early stroke detection and prediction by analyzing its capacity to identify subtle imaging markers and enhance diagnostic precision in the critical early stages. The most compelling current research hotspots are the optimal selection of neural imaging markers and the most suitable ML algorithm models for these purposes. In the future, researchers can continue to develop high-performance algorithms, further advancing early diagnosis and personalized treatment strategies in this scientific domain.

### Limitations

This study is subject to several limitations. Firstly, it primarily relies on data accessible within the WOSCC database. CiteSpace is incapable of integrating data from varied databases or performing citation analysis on sources outside of WOSCC. Secondly, while CiteSpace proves invaluable in detecting and visualizing emerging trends, it does not delve into the underlying mechanisms of machine algorithm models in the application of identifying MRI features for early stroke diagnosis. Despite these constraints, we have successfully employed CiteSpace to illustrate the latest research trends in the application of machine algorithm models in the early diagnosis of stroke through the recognition of MRI characteristics.

## Data Availability

The original contributions presented in the study are included in the article/supplementary material, further inquiries can be directed to the corresponding author.
